# Implantable venous access port catheter fracture in a severely obese lung cancer patient undergoing chemotherapy: a case report

**DOI:** 10.3389/fonc.2026.1835632

**Published:** 2026-06-18

**Authors:** Tingting Liu, Hu Luo, Qiangzhong Pi, Xiaoling Wu, Jiasi Zhang, Jing Deng

**Affiliations:** 1 Department of Respiratory and Critical Care Medicine, First Affiliated Hospital of Army Medical University (Southwest Hospital), Chongqing, China; 2 Department of Hematopathology, First Affiliated Hospital of Army Medical University (Southwest Hospital), Chongqing, China

**Keywords:** case report, chemotherapy complication, implantable venous access port, obesity, port fracture

## Abstract

While implantable venous access ports (IVAPs) are a cornerstone of chemotherapy administration, they carry the risk of mechanical failures, including catheter fracture. We describe a rare instance in a 33-year-old patient with lung cancer and class III obesity (BMI 42.1 kg/m^2^), who developed episodic pain around the port pocket. Diagnostic imaging revealed a structural failure of the catheter inside the port mechanism. A coordinated multidisciplinary effort ensured prompt retrieval of the device, averting potential migration. This report suggests that extreme obesity may exacerbate mechanical stress on IVAPs, necessitating enhanced surveillance for patients with high BMI.

## Introduction

Lung cancer remains the leading cause of cancer-related mortality in China. With over 4.5 million new cancer diagnoses reported in 2020, lung malignancies comprise a significant portion of the disease burden (1, 2). Since systemic chemotherapy is the standard of care, reliable long- term vascular access is critical. Peripheral venous catheters, while accessible, are ill-suited for prolonged regimens due to the cumulative risk of phlebitis, endothelial damage, and thrombosis, which can compromise patient compliance and quality of life.

Totally implantable venous access devices (TIVADs) have become the preferred alternative, offering reduced infection rates and minimizing daily interference with patients’ lives compared to external central lines (3–5). Consequently, they are widely adopted for patients requiring intermittent, long-term infusion therapy (6, 7), such as cancer chemotherapy patients, serving as a safe, reliable, and enduring “lifeline” supporting their continuous treatment.

Despite their advantages, TIVADs are subject to mechanical complications, ranging from occlusion to rare but catastrophic catheter fractures (5, 8).Catheter embolization poses severe risks, including arrhythmia and pulmonary embolism (9, 10).While mechanisms such as the “pinch-off syndrome” (clavicular compression) are well-documented, the impact of patient body habitus—specifically severe obesity—on device durability and diagnosis remains underreported in China (11, 12). The thick subcutaneous adipose tissue in obese patients not only complicates insertion but may also obscure early signs of mechanical failure.

We herein report a case of catheter fracture in a 33-year-old patient with class III obesity. This case illustrates how extreme body mass index (BMI) can mask physical signs of catheter disconnection and discusses the implications for surveillance in this specific patient population.

This case highlights the critical importance of strict adherence to standard operating procedures and individualized assessment during IVAP placement for patients with unique somatotypes. Monitoring and comprehensive management are essential to prevent such complications, thereby providing valuable clinical insights for the early management of catheter fractures.

## Case history/examination

This study was approved by the Institutional Review Board (Approval No. (B2)2025KY054).

A 33-year-old man with a BMI of 50.8 kg/m^2^ presented with a progressive respiratory illness beginning in 2021 after cold exposure, starting as paroxysmal cough with easily expectorated white frothy sputum. He later developed small-volume, blood-streaked sputum but did not seek care. After partial symptomatic improvement with oral antibiotics in January 2022, his cough recurred and worsened by mid-2022, especially when supine, with paroxysmal yellowish-white sputum and exertional dyspnea; nocturnal dyspnea occurred without fever or chest pain.

A chest CT performed on April 29, 2022, indicated: a possible new growth near the left hilum with obstructive atelectasis; a tiny ground-glass nodule in the right lower lobe superior segment, likely inflammatory; and enlarged mediastinal lymph nodes. On May 22, 2022, a bronchoscopy with biopsy was performed. Postoperative pathology revealed: (bronchoscopic biopsy specimen) small cell lung cancer. PET/CT performed on May 26, 2022, showed: 1. A mass at the left hilum with increased FDG uptake, consistent with lung cancer based on pathology; 2. Enlarged left supraclavicular and mediastinal lymph nodes with increased FDG uptake, suggestive of metastasis. Chemotherapy and immunotherapy were initiated on June 1, 2022. Due to the vesicant nature of the required chemotherapy and the need for cyclic treatment every 21 days, an implantable venous port was selected to ensure secure, long-term access. Between early October and mid-November 2022, the patient underwent thoracic lesion radiotherapy for a total of 28 sessions (**Table 1**). The patient has no history of arm or chest trauma or surgery.

**Table 1:** Timeline and treatment methods of patients.

Timeline	Chemotherapy	Immunotherapy	Radiotherapy
June 1st, 2022	Etoposide 0.15g D1-5 + Carboplatin 1000mg D1		
June 6th, 2022		Atelizumab 1200mg	
June 30th, 2022		Dovacetuzumab 1500mg	
July 1st, 2022	Etoposide 0.1g D1-5 + Carboplatin 800mg D1		
July 27th, 2022		Dovacetuzumab 1500mg	
July 28th, 2022	Etoposide 0.1g D1-5 + Carboplatin 800mg D1		
August 26th, 2022		Dovacetuzumab 1500mg	
August 27th, 2022	Etoposide 0.1g D1-5+ Carboplatin 800mg D1		
September 23th, 2022	Etoposide (Vorinostat) 0.15g D1-2 + Etoposide (Vorinostat) 0.1g D3-5 + Carboplatin 800mg D1	Dovacetuzumab 1500mg	
October-November, 2022			28 sessions of chest radiotherapy with IMRT (intensity-modulated radiotherapy, 28 sessions, 50.4 Gy, 1.8 Gy per session)
January 4th, 2023	Irinotecan Hydrochloride (Aili) 480mg + Lomustine 75mg D1		
February 14th, 2023	Irinotecan Hydrochloride (Aili) 320mg D1 + Lomustine 100mg D1	Dovacetuzumab 1500mg	
March 23th, 2023	Paclitaxel Liposome (Fupusun) 360mg + Nedaplatin (Oxacin 10mg) 170mg	Dovacetuzumab 1500mg	
April 19th, 2023	Paclitaxel Liposome (Fupusun) 360mg + Nedaplatin (Oxacin 10mg) 170mg	Dovacetuzumab 1500mg	
June 6th, 2023		Dovacetuzumab 1500mg	
June 9th, 2023	Paclitaxel Liposome (Fupusun) 360mg + Nedaplatin (Oxacin 10mg) 170mg		
July 19th, 2023	Paclitaxel Liposome (Fupusun) 360mg + Nedaplatin (Oxacin 10mg) 170mg		
August 16th, 2023		Dovacetuzumab 1500mg	
September 8th, 2023	Paclitaxel Liposome (Fupusun) 360mg + Nedaplatin (Oxacin 10mg) 170mg	Dovacetuzumab 1500mg	
October 12th, 2023		Dovacetuzumab 1500mg	
November 6th, 2023	Paclitaxel (Albumin-Bound Type) 600mg + Nedaplatin (Onsorad 10mg) 170mg	Dovacetuzumab 1500mg	
December 1st, 2023	Paclitaxel (Albumin-Bound Type) 600mg + Nedaplatin (Onsorad 10mg) 170mg	Dovacetuzumab 1500mg	
December 29th, 2023	Paclitaxel (Albumin-Bound Type) 600mg + Nedaplatin (Onsorad 10mg) 170mg	Dovacetuzumab 1500mg	
February 2nd, 2024	Paclitaxel (Albumin-Bound Type) 600mg + Nedaplatin (Onsorad 10mg) 170mg	Dovacetuzumab 1500mg	
March 8th, 2024		Dovacetuzumab 1500mg	
March 29th, 2024	Paclitaxel (Albumin-Bound Type) 600mg + Nedaplatin (Onsorad 10mg) 160mg	Dovacetuzumab 1500mg	
May 14th, 2024		Dovacetuzumab 1500mg	
June 12th, 2024		Dovacetuzumab 1500mg	
July 20th, 2024	Paclitaxel (Albumin-Bound Type) 500mg + Nedaplatin (Onsorad 10mg) 140mg	Dovacetuzumab 1500mg	
August 28th, 2024		Dovacetuzumab 1500mg	
September 27th, 2024		Dovacetuzumab 1500mg	
November 12th, 2024		Dovacetuzumab 1500mg	

**Figure 1:**
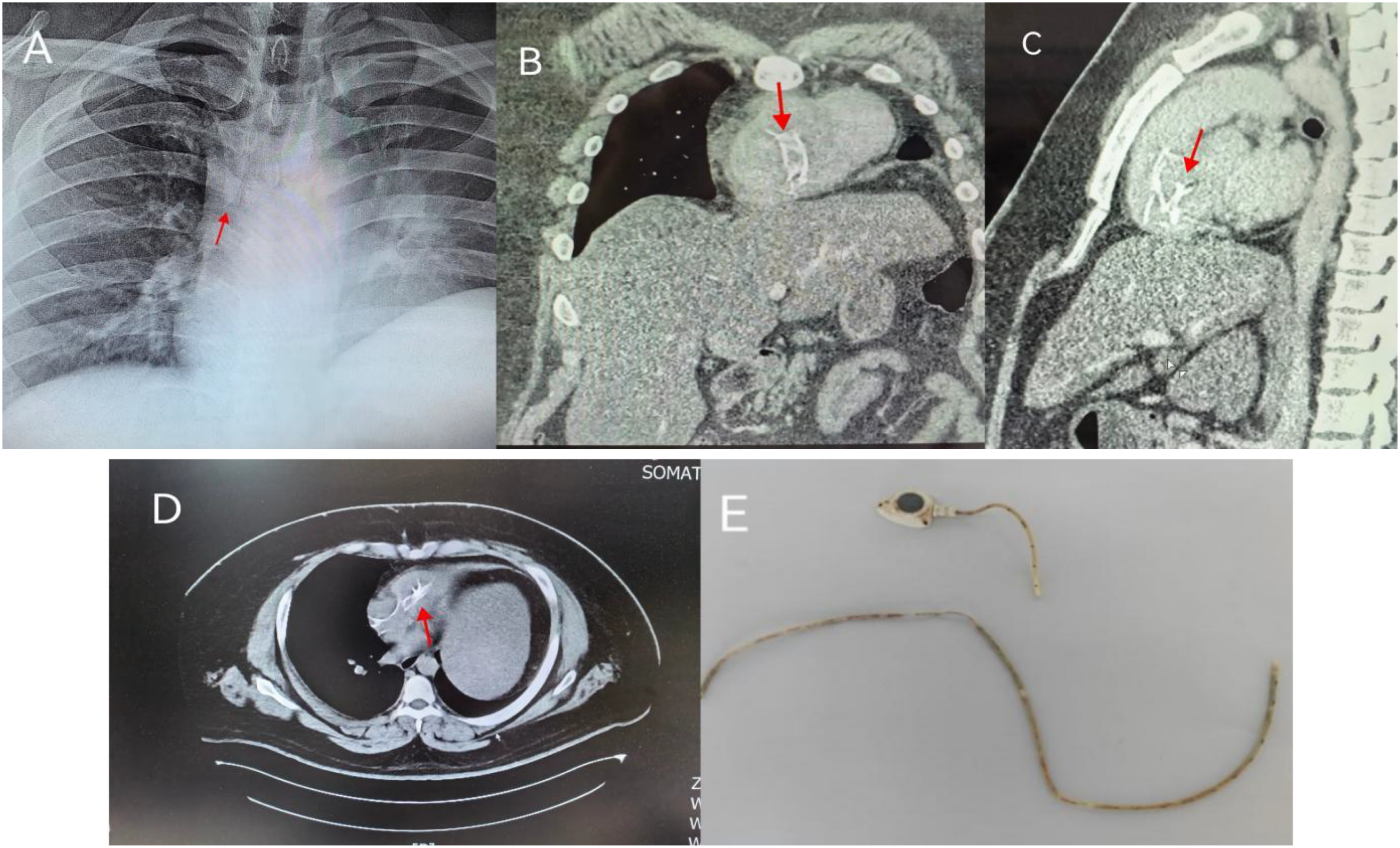
**(A)** The position of the intravenous infusion port catheter tip after insertion. **(B–D)** An image showing suspected displacement of the tip of the implanted venous infusion port to the right atrium. **(E)** Remove the fractured port base and catheter of the implantable venous infusion port.

On June 30, 2022, a totally implantable venous access port (TIVAP) was placed by a respiratory physician and an intravenous therapy specialist nurse, both of whom were certified in arm port implantation with over five years of relevant clinical experience. Under ultrasound guidance, a polyurethane port (Suzhou Linhua Medical Instrument Co., Ltd., Jiangsu, China) was implanted via the right basilic vein. The inserted catheter length was 39 cm, and the patient’s right upper arm circumference was 45 cm. The lengths of the subcutaneous tunnel and the port pocket were approximately 2.0 cm and 2.0 cm, respectively. The procedure was uneventful. Following the connection of the catheter to the port chamber and its placement into the subcutaneous pocket, adequate blood return was confirmed. The incision was closed in layers using interrupted silk sutures, ensuring optimal skin approximation. After iodophor disinfection, a sterile dressing was applied securely with no signs of immediate bleeding or exudation. Postoperative access using a non-coring needle demonstrated excellent blood return and unobstructed flushing. The patient reported no postoperative discomfort. A post-procedural chest radiograph verified the optimal positioning of the catheter tip at the level of the fifth thoracic vertebra (**Figure 1A**).

In early December 2024, the patient reported intermittent pain at the port implantation site. During a follow-up visit to the intravenous therapy clinic on December 10, 2024, blood could not be aspirated from the port despite positional adjustments. Subsequent attempts to flush the catheter with normal saline elicited ipsilateral arm pain, prompting immediate cessation of the procedure. The patient was admitted on the same day for further evaluation and management of the port dysfunction. Since the onset of these symptoms, the patient has remained hemodynamically stable and alert, reporting normal appetite, sleep, and bowel/bladder functions, without significant weight loss. His medical and family histories were unremarkable for genetic disorders, and his psychosocial baseline was stable.

Venous blood analysis revealed elevated liver enzymes: alanine aminotransferase (ALT) at 120 IU/L (reference range: 0–42 IU/L), aspartate aminotransferase (AST) at 92.3 IU/L (reference range: 0–42 IU/L), and gamma-glutamyl transferase (GGT) at 595.3 IU/L (reference range: 4–50 IU/L). All other laboratory parameters were within normal limits.

## Methods

### Differential diagnosis

The etiology of pain at an implanted port access site broadly includes:mechanical complications such as catheter or port damage, infection or inflammatory response, with local infection presenting as redness, swelling, heat, and pain, and individual factors such as thin subcutaneous fat, hypersensitive skin, or anxiety reducing pain tolerance.

### Final diagnosis

The patient presented without fever or visible signs of inflammation like redness or heat. We could feel the port body but not the catheter itself. When we tried to use the port, there was no blood return, and flushing with saline caused pain. We stopped immediately. No swelling was seen, but this was likely due to the patient’s size. We admitted him to determine the cause of the dysfunction.

Initial chest radiography and computed tomography (CT) scans revealed the absence of the catheter along its original venous course and identified a radiopaque linear structure within the right atrium and ventricle, indicating catheter fracture and migration (**Figures 1B–D**).

To address this, surgical exploration of the port pocket was performed under local anesthesia at 18:27 on December 11, 2024. An approximately 3-cm incision was made adjacent to the original reservoir site, followed by coarse dissection through the fascial layer. The reservoir was extracted intact with minimal blood loss (approximately 10 mL); however, only a 7.5-cm segment of the proximal catheter remained attached. Given the original implanted length of 39 cm, the residual 31.5-cm distal fragment was presumed to have embolized into the central venous system. The incision was irrigated, closed primarily, and sterilely dressed. The patient was instructed on wound care and prescribed oral cefdinir for antimicrobial prophylaxis.

To precisely localize the migrated fragment for subsequent retrieval, an emergency contrast-enhanced chest and abdominal CT was performed at 20:40 the same day. The imaging confirmed a high-density foreign body traversing the right atrium and right ventricle, consistent with the embolized catheter fragment. The primary physician promptly arranged echocardiography and convened an urgent multidisciplinary team (MDT) consultation, comprising specialists from respiratory medicine, vascular surgery, cardiac surgery, and interventional radiology. The consensus was to proceed with percutaneous endovascular retrieval.

At 21:40, the patient was transferred to the interventional suite. Following angiography of the inferior vena cava, right atrium, and right ventricle to map the anatomy, endovascular retrieval was successfully performed. The extracted catheter fragment was entirely intact and measured exactly 31.5 cm (**Figure 1E**), which mathematically accounted for the total original length of 39 cm. An EXOSEAL vascular closure device (EX700, Cordis) was deployed to seal the puncture site, followed by a pressure dressing. The entire procedure was uneventful and well-tolerated by the patient, with vital signs remaining stable throughout.

## Conclusion and results (outcome and follow-up)

Postoperatively, a pressure dressing was applied to the venous access site for 6 to 8 hours to prevent hematoma formation. The patient was transferred to the Respiratory Intensive Care Unit (RICU) for close monitoring, which included serial assessments of the ipsilateral dorsalis pedis pulse and distal extremity perfusion. The postoperative course was uncomplicated, with no evidence of hemorrhage or vascular compromise. Consequently, the patient was stepped down to the general respiratory ward the following day (December 12).

For subsequent pharmacological management, a peripheral intravenous line was established in the left upper extremity to administer a 10-day course of hepatoprotective therapy (glutathione, 1.8 g). On December 17, a temporary central venous catheter (CVC) was inserted via the internal jugular vein to facilitate a 3-day chemotherapy regimen (etoposide, 0.1 g, days 1–3). Following the removal of the fractured TIVAP and the endovascular retrieval procedure, the patient was initiated on subcutaneous enoxaparin (40 mg/0.4 mL, twice daily) for thromboprophylaxis. In preparation for discharge, the anticoagulation regimen was transitioned to oral rivaroxaban (10 mg, once daily) on December 22. After an uncomplicated observation period, the patient was officially discharged on December 23, 2024.

During the follow-up period up to October 27, 2025, the patient underwent two additional chemotherapy cycles (March 7 and July 4, 2025) via temporary internal jugular CVCs, both of which were removed immediately after completion of the infusions. Additionally, he completed five cycles of immunotherapy. Throughout the follow-up duration, no delayed intervention-related complications were observed, and the patient’s clinical outcomes remained favorable.

## Discussion

Although implantable venous access ports offer improved quality of life and cosmetic benefits, mechanical failure remains a critical, life-threatening complication. Catheter fracture is particularly insidious as it may manifest with vague symptoms before leading to catastrophic embolism. In clinical practice, the ‘pinch-off’ sign or catheter disconnection is typically screened via palpation. However, as demonstrated in this case, obesity limits the utility of physical examination. Assessment must therefore rely on functional checks (patency, resistance) and a low threshold for imaging if patient discomfort arises.

This report highlights a gap in the literature regarding catheter fatigue in obese patients. The catheterization operation in this study was jointly completed by respiratory physicians and respiratory nurses who held certificates for upper arm infusion port placement and specialized nurse certificates in venous therapy, and had more than 5 years of relevant experience. The procedure was performed under ultrasound guidance through the brachial vein of the right arm. The product used was a common venous infusion port product. The operation process followed the standard procedures. The entire process was smooth. During the operation, the blood was successfully withdrawn and the flushing was unobstructed. Postoperatively, a chest X-ray confirmed that the tip of the catheter was in the normal position. The patient reported no discomfort.

Our patient, who utilized the device for 30 months, suffered an intravascular fracture likely driven by chronic mechanical stress. The patient’s habit of sleeping in the right lateral decubitus position, combined with increased tissue pressure from obesity, likely accelerated material fatigue while simultaneously masking the physical signs of disconnection.

Concurrently, the sustained compression from the patient’s sleeping posture may have accelerated material fatigue. Studies indicate that polyurethane-type catheters can undergo age-related degradation, reducing tensile strength over time (13), which aligns with previous case reports (14). Procedural factors—such as catheter clamping, excessive traction during insertion, or forced flushing against resistance during maintenance (15)—may also contribute to catheter damage. Although this patient underwent 28 rounds of radiotherapy, radiotherapy may have potential indirect effects, such as causing local tissue fibrosis, changes in the vascular wall, or affecting surrounding anatomical structures, which may potentially increase the mechanical environmental pressure on the catheter. However, this is only a hypothesis and more research is needed to confirm this.

Early-phase catheter disruptions, given their frequent asymptomatic nature make clinical detection challenging. A systematic, multi-level prevention strategy is essential. At the clinical practitioner level, strict adherence to standardized catheter placement techniques is required, employing coarse separation methods to minimize mechanical damage and selecting catheter materials appropriately based on individual patient characteristics. Prior to using an infusion port, a systematic assessment protocol must be performed: initially assess catheter continuity via palpation (though this is limited in obese patients), followed by blood aspiration and saline injection using a dedicated non-damaging needle to observe for warning signs such as resistance, pain, localized swelling, or abnormal blood return. Any abnormalities necessitate immediate imaging studies (e.g., chest X-ray) to confirm catheter status. Additionally, establish a regular assessment protocol, such as a comprehensive “aspiration-injection-palpation-questioning” check every 7 days, combined with imaging to dynamically monitor catheter position and structural integrity.

Regarding patient education, emphasize the importance of daily self-management. This includes avoiding vigorous activities, heavy lifting, or pressure on the catheterized limb; maintaining a healthy weight to reduce mechanical load; and regularly monitoring the puncture site for redness, swelling, pain, or other abnormalities. Patients should also learn basic emergency procedures, such as immediately lying flat and seeking medical attention if discomfort arises. Through collaborative doctor-patient efforts and standardized management, complication risks can be significantly reduced, ensuring treatment safety.

## Limitations

Biomechanical analysis and material testing of the fractured segment were not performed, preventing a quantitative assessment of stress from compression and fatigue. Long-term data on the efficacy of posture modification are also lacking. Future studies should explore the relationship between obesity, sleeping positions, and catheter failure. Improved monitoring protocols are needed for high-risk patients, especially those who are obese or have specific positional needs.

## Conclusion

We reported a case of catheter fracture in a lung cancer patient presenting with localized port site pain. Prompt identification and multidisciplinary collaboration allowed for the successful retrieval of the embolized fragment without complications. This case underscores the critical need for heightened clinical vigilance in vulnerable populations, particularly when physical assessment is limited by patient body habitus.

## Data Availability

The original contributions presented in the study are included in the article/supplementary material. Further inquiries can be directed to the corresponding author.

## References

[1] Qiu HB Cao SM Xu RH . Time trends of cancer incidence, mortality and burden in China based on global epidemiological data analysis in 2020 and comparison with data from the United States and the United Kingdom. Cancer. (2022) 41:165–77. doi: 10.1002/cac2.12197 34288593 PMC8504144

[2] The health management branch of the Chinese medical association The radiology branch of the Chinese medical association The editorial committee of "Chinese Journal of Health Management . Expert consensus on lung cancer screening and pulmonary nodule health management (2025 Edition). Chin J Health Manag. (2025) 19:759–69. doi: 10.3760/cma.j.cn115624-20250828-00731 30704229

[3] Zhou Y Lan Y Zhang Q Song J He J Peng N . Totally implantable venous access ports: A systematic review and meta-analysis comparing subclavian and internal jugular vein punctures. Phlebology. (2022) 37:279–88. doi: 10.1177/02683555211069772 35200052

[4] Salawu K Arowojolu O Afolaranmi O Jimoh M Nworgu C Falase B . Totally implantable venous access ports and associated complications in sub-Saharan Africa: A single-centre retrospective analysis. Ecancermedicalscience. (2022) 16:1389. doi: 10.3332/ecancer.2022.1389 35919223 PMC9300414

[5] Hara Y Sumida Y Yamazaki S Takei D Yamashita M Fukuda A . Risk factors for infection of totally implantable central venous access ports among patients requiring port removal. J Vasc Access. (2025) 26:519–24. doi: 10.1177/11297298231225808 38316617

[6] Yu Z Sun X Bai X Ding W Wang W Xu L . Perioperative and postoperative complications of supraclavicular, ultrasound-guided, totally implantable venous access port via the brachiocephalic vein in adult patients: A retrospective multicentre study. Ther Clin Risk Manag. (2021) 17:137–44. doi: 10.2147/TCRM.S292230 33568912 PMC7869700

[7] Liu C Liu X Zhao S Li W . Port-exposure management of totally implantable venous access ports: A case report. J Cancer Res Ther. (2023) 19:1064–9. doi: 10.4103/jcrt.jcrt_666_23 37675738

[8] Huang XM Li X Deng J Chen J Qian L . Clinical applications and research progress of totally implantable venous access ports: A literature review. Front Oncol. (2025) 14:1519728. doi: 10.3389/fonc.2024.1519728 39886665 PMC11779708

[9] Yuan Z Chen YY Li XY Lin Q Xia KP . Treatment and reflection of a case of complete rupture in an implanted intravenous infusion port under multidisciplinary cooperation. Chin J Pract Nurs. (2019) 35:2031–4.

[10] Li Y Han N Wang XX Li HN Liang X . Analysis on 13 cases with rupture of venous port access *in vivo*. Chin J Mod Nurs. (2018) 24:833–5. doi: 10.3760/cma.j.issn.1674-2907.2018.07.022 30704229

[11] Tao J Li ZL Zhang CY Yan WP Luo CG . One case: Venous access port catheter disconnection and gone off caused by catheter lock fracture. J Pract Radiol. (2023) 39:1905–6. doi: 10.3969/j.issn.1002-1671.2023.11.043

[12] Yuan JB . Prevention and management of implantable venous port catheter fracture. Electron J Clin Med Lit. (2020) 7:117. doi: 10.16281/j.cnki.jocml.2020.08.102

[13] Guerreiro H Schröder H Huber G Busch F Sellenschloh K Adam G . Quantification of mechanical properties in long-term *in vivo* used silicone catheter lines according to DIN 10555-3. Clin Biomech (Bristol). (2023) 107:106015. doi: 10.1016/j.clinbiomech.2023.106015 37321163

[14] Kondo T Matsumoto S Doi K Nomura M Muto M . Femoral placement of a totally implantable venous access port with spontaneous catheter fracture: Case report. CVIR Endovasc. (2020) 3:2. doi: 10.1186/s42155-019-0094-9 32027011 PMC6966363

[15] Wang JX Wu ZL Su JN Xie YL Sun YQ Jiao JQ . Diagnosis and treatment of the cause of catheter dysfunction of central verlous port access system. J Hebei Med Univ. (2016) 37:1192–5. doi: 10.3969/j.issn.1007-3205.2016.10.020

